# TP53-mutated myelodysplastic syndromes and acute myeloid leukemia: a comprehensive overview of targeted approaches

**DOI:** 10.3389/fonc.2026.1735418

**Published:** 2026-02-23

**Authors:** Shweta Deshpande, Wing Fai Li, Junmin Song, Mark Forsberg, Claudio Cerchione, Giovanni Martinelli, Marina Konopleva

**Affiliations:** 1 Department of Oncology, Montefiore Einstein Comprehensive Cancer Center, Bronx, NY, United States; 2 Department of Medicine, Jacobi Medical Center, Albert Einstein College of Medicine, Bronx, NY, United States; 3 Department of Hematology and Oncology, IRST IRCCS, Meldola, Italy; 4 Department of Medical and Surgical Sciences, Università di Bologna, Bologna, Italy; 5 Department of Cell Biology, Albert Einstein College of Medicine, Bronx, NY, United States

**Keywords:** acute myeloid leukemia, myelodysplastic syndromes, TP53 mutation, p53, hypomethylating agents, venetoclax, allogeneic stem cell transplantation

## Abstract

TP53-mutated acute myeloid leukemia (AML) and myelodysplastic syndromes (MDS) represent a biologically and clinically distinct subset of myeloid malignancies characterized by poor prognosis, resistance to standard therapies, and high rates of relapse. TP53 mutations, particularly biallelic are frequently associated with complex karyotypes and confer profound chemoresistance. Although hypomethylating agents and venetoclax-based combinations provide modest benefit, durable remissions remain rare. Novel therapeutic strategies targeting mutant p53, restoring wild-type function, or exploiting synthetic lethal pathways are under active investigation. This review aims to summarize current knowledge on the biology of TP53, prognostic implications, and therapeutic landscape of TP53-mutated AML/MDS, ongoing and past clinical trials in TP53-mutated AML/MDS patients, emphasizing the need for precision-guided, multimodal approaches to improve outcomes in this high-risk group.

## Introduction

Myelodysplastic syndrome (MDS) and acute myeloid leukemia (AML) are a group of clonal hematological malignancies marked by ineffective blood cell production, cytopenia, and the accumulation of immature myeloid blasts in the bone marrow and peripheral circulation. AML is the most prevalent form of acute leukemia in adults. In the United States, the median age of AML diagnosis is approximately 68 years, and its incidence is increasing with age (1). In 2025, there were an estimated 22,010 new cases of AML in the United States (2). Although treatment outcomes for AML have improved over the years, survival disparities between younger and older patients remain striking. Based on SEER registry data from 2001–2018, among chemotherapy-treated adults, 5-year survival reached 59.1% in those aged 20–39 and 42.6% in those 40–59, but declined sharply to 21.0% for ages 60–74 and fell below 6% for patients ≥75 years (1). This limited improvement is largely due to poor tolerance of intensive standard induction, such as the standard cytarabine and anthracycline-based chemotherapy (7+3) regimen, and the higher prevalence of adverse genetic mutations in older patients (3).

Advances in next-generation sequencing (NGS) technology have deepened our insights into the genetic landscapes of AML and MDS, leading to the identification of precursor conditions. For instance, clonal hematopoiesis of indeterminate (CHIP) or oncogenic potential (CHOP) are now recognized as early events in leukemogenesis. Successive WHO classifications have progressively incorporated molecular and cytogenetic features into the diagnostic framework of myeloid neoplasms, with the 2022 WHO and ICC classifications further refining disease categorization by formally recognizing TP53-mutated myeloid neoplasms as a distinct high-risk entity (4, 5). A notable change includes the distinct classification of TP53-mutated neoplasms, defined by at least one somatic TP53 mutation with a variant allele frequency (VAF) above 10%. This change reflects the consistently poor prognosis associated with TP53 mutations (6–8) and their tendency to coincide with complex karyotype or therapy related AML (tAML) (3).

The TP53 gene, located on the chromosome 17p13.1, encodes the p53 protein, a key transcription factor that responds to cellular stress, including DNA damage, oncogene activation, and hypoxia (9). Upon activation, p53 promotes DNA repair, induces apoptosis, and regulates cell cycle progression and cellular differentiation (10–13). TP53 mutation (TP53m) is one of the most common genetic alterations across all human cancers. In AML, TP53 mutations are observed in approximately 5-15% of *de novo* cases and 15-30% of secondary or therapy-related AML, reflecting inter-study variability (14–17). Such mutations correlate with unfavorable clinical outcomes, driving primary resistance to chemotherapy, and an overall survival of less than 1 year (14, 18, 19). The tumor-suppressive function of p53 is essential for effective apoptosis in response to cytotoxic therapy; thus, TP53 mutations confer intrinsic resistance to conventional chemotherapy regimens.

The management of TP53-mutated MDS and AML remains a significant clinical challenge. Despite progress in understanding its molecular underpinnings, TP53 remains one of the most difficult targets in cancer biology. This review explores the molecular biology of TP53 mutations, their prognostic implications, and the efficacy of current and investigational treatment strategies aimed at overcoming resistance in this high-risk patient population.

## Methodology

This narrative review is based on a literature search of PubMed/MEDLINE, ClinicalTrials.gov and major hematology conference abstracts (ASH, EHA) covering publications from January 2000 through March 2025. Studies were selected based on relevance to TP53-mutated AML and MDS, including biological studies, prognostic analyses, and clinical trials.

### TP53 gene biology, physiologic functions and mutation in AML

#### Biology

**Figure 1:**
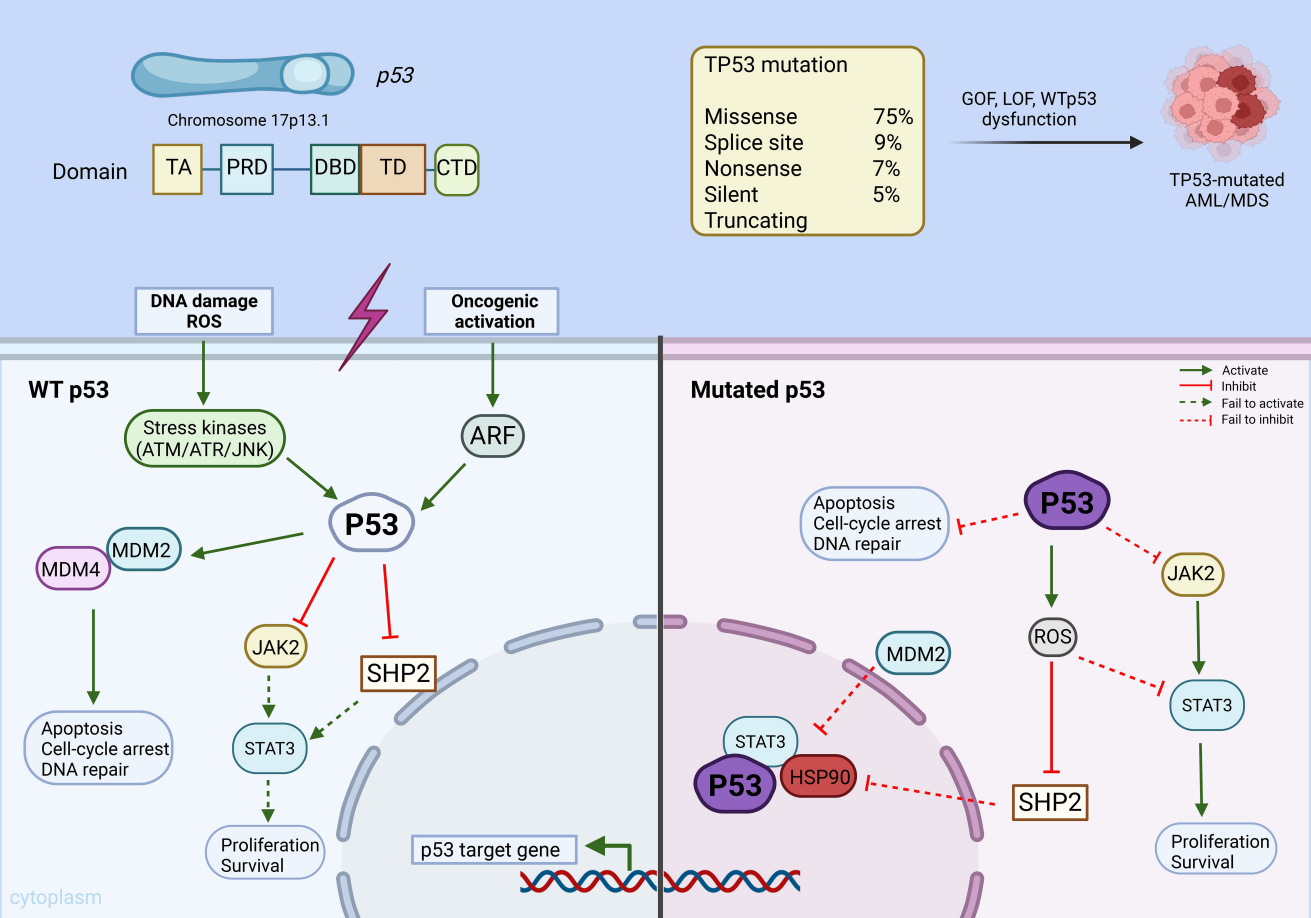
A simplified overview of p53 biology and its regulation in cells with wild-type p53 and mutated p53. Under cellular stress (e.g. DNA damage, oncogene activation, or reactive oxygen species), upstream stress kinases such as ATM, ATR, and JNK phosphorylate p53, disrupting its interaction with negative regulators MDM2 and MDM4. Activated ARF from oncogenic stress binds MDM2 and inhibits it from ubiquitylating p53. Stabilized p53 accumulates in the nucleus to activate transcription of p53 target genes that enforce cell-cycle arrest, trigger apoptosis, and facilitate DNA repair. Wild-type p53 also restrains proliferative/survival signaling by antagonizing the JAK2–STAT3 pathway, in part through induction or activation of negative regulators such as SHP2, thereby reinforcing its tumor-suppressive function. On the other hand, mutant p53 proteins often evade degradation partly through aberrant interactions with molecular chaperones like HSP90. MDM2 is unable to target mutant p53 proteins for degradation. As a result, mutant p53 fails to induce cell-cycle arrest, apoptosis, or DNA repair. Mutant p53–associated dysregulation also leads to ROS accumulation and subsequently impaired SHP2 activity, collectively promoting leukemic cell proliferation and survival. ROS: reactive oxygen species, ATM: ataxia telangiectasia mutated, ATR: ataxia telangiectasia and Rad3-related protein, JNK: c-Jun N-terminal kinase, ARF: alternate reading frame protein, MDM2: mouse double minute 2 homolog, MDM4 (MDMX): MDM4 homolog, SHP2: Src homology region 2 domain-containing phosphatase-2, JAK2: Janus kinase 2, STAT3: signal transducer and activator of transcription 3, HSP90: heat shock protein 90, LOF: loss-of-function, GOF: gain-of-function. Created with BioRender.com.

At chromosomal locus 17p13.1, TP53 gene encodes the wild-type p53 transcription factor, which is vital for orchestrating stress signaling and preserving genome stability. The p53 protein product has five functional regions: (a) the amino N-terminal transactivation domain (TA), (b) the proline-rich domain (PRD), (c) the central DNA-binding domain (DBD), (d) the tetramerization domain (TD), and (e) carboxy-terminal oligomerization domain (CTD) at the C-terminus (11, 20–22). (**Figure 1**) This tetrameric structure facilitates the sequential binding of p53 to various cofactors following stress signals such as DNA damage, replication errors or oncogene activation (13). Upon cellular stress, p53 coordinates cell cycle regulation, promotes DNA repair, and induces apoptosis, thus functioning as a critical safeguard against malignant transformation (23, 24). TP53 mutations impair the specific binding of the protein to its cofactors, leading to tumor initiation, proliferation and chemoresistance (25). These changes are predominantly attributed to missense mutations causing an amino acid change in the DBD (~75%) (9, 26, 27) with splice-site indels accounting for 9%, nonsense mutations for 7%, silent changes for 5%, and other truncating variants making up the remainder (28, 29). The mutations can cause proliferation of tumor cells via gain of function (GOF), a loss of function (LOF) or WTp53 dysfunction (13).

#### The p53 pathway

Under normal physiological conditions, p53 functions as a transcription factor with a short half-life and remains largely inactive due to tight regulation by the negative regulators MDM2 (Mouse Double Minute 2) and MDM4 (also known as MDMX). These proteins promote ubiquitination and subsequent proteasomal degradation of p53, maintaining its low basal levels in unstressed cells (30–32). However, upon exposure to cellular stress, such as DNA damage or oncogenic signaling (33), the p53/MDM2 interaction is disrupted leading to increase in intracellular p53 stabilization. This stabilization is further enhanced through post-translational modifications including phosphorylation, methylation and acetylation (34–38). The stabilized p53 either induces cell differentiation or activates transcription of genes leading to apoptosis and cell cycle arrest in cells with mutated or damaged DNA. Interestingly, wild-type p53 (WTp53) may be rendered dysfunctional in some cancers, including primary AML, due to overexpression of MDM2 and/or MDM4 (MDMX), which suppresses p53 activity and is associated with inferior outcomes (11). The tumor suppressor P14/ARF positively regulates p53 by inhibiting MDM2, thus preventing p53 degradation and promoting its stabilization. Low P14ARF expression has been linked to poor prognosis in several studies (39–41).

#### TP53 mutation in MDS/AML

TP53 mutations appear in only 5–10% of *de novo* MDS/AML cases but are far more frequent in secondary AML (18%) and therapy-related AML (30%) (42–44). These mutations are often associated with complex karyotypes (CK) and monosomal karyotypes (MK) (8, 45–47), both indicative of genomic instability. TP53 mutations in MDS/AML can be monoallelic or biallelic (48). In this review, we use the term multihit TP53 as an umbrella concept encompassing classical biallelic alterations as well as functional equivalents, including multiple TP53 mutations, high variant allele frequency suggestive of copy-neutral loss of heterozygosity, or cytogenetic loss of chromosome 17p. The term biallelic TP53 is used when structural involvement of both alleles can be directly inferred. This terminology is consistent with contemporary WHO and ICC frameworks and reflects current clinical practice. Approximately 70% of the cases exhibit biallelic alterations, which typically involve a point mutation in one allele and the loss of the second allele through 17p deletion or monosomy 17. In contrast, monoallelic mutations (25-30%) usually consist of a single point mutation, frequently observed in patients with isolated 5q deletion MDS (22, 49–51). TP53 mutations may also co-occur with other mutations, commonly in TET2 (29%), SF3B1 (27%), ASXL1 (16%), and DNMT3A (16%) which can influence disease progression and treatment outcomes (48). Furthermore, chromothripsis or chromosome shattering (chromosomal rearrangement) (52) is a phenomenon involving catastrophic chromosomal rearrangements which can lead to loss of heterozygosity by inactivating the remaining TP53 allele, particularly in cases with 17p deletion (53). This process is closely linked to TP53 mutations, cell cycle dysfunction, and poor prognosis in AML patients featuring complex karyotype (53). It has been recognized that biallelic or “multihit” involvement with multiple mutations or loss of heterozygosity is seen in 44% of MDS patients with TP53 mutations (48). Conversely, in low-risk MDS, about 20% of patients harbor monoallelic mutations alongside 5q deletion. These cases tend to occur in younger individuals and have a less aggressive clinical course than biallelic mutations (54).

The prognostic and therapeutic impact of TP53 allelic status has emerged as a key modifier of response. In MDS, patients with monoallelic TP53 mutations-defined as a single mutation without 17p deletion or replacement of the allele with its mutant counterpart (termed copy neutral loss of heterozygosity or CN-LOH)-demonstrate outcomes comparable to TP53 wild-type disease, with a median overall survival of ~2.5 years and 5-year OS around 40% (48). In contrast, multi-hit TP53 alterations, characterized by loss of the second allele via deletion, CN-LOH, or a second mutation, are strongly associated with complex karyotype and poor prognosis, with a median OS of only 8.7 months and 5-year OS consistently <10%, even after intensive chemotherapy or transplant (48).

Importantly, standard molecular testing for myeloid malignancies such as NGS gene panels and cytogenetic analyses may fail to detect CN-LOH. To accurately assess TP53-mutant allele status, specialized methods capable of identifying CN-LOH are required. These include expanded gene panels specifically designed to detect TP53 LOH, single-nucleotide polymorphism (SNP) arrays, or whole-genome/exome sequencing (55, 56). Detecting a single TP53 mutation having a VAF of greater than or equal to 50% is considered to be presumptive evidence of loss of the wild-type allele or CN-LOH. However, some cases of CN-LOH with low leukemia burden could be missed (55, 57).

Precisely distinguishing between monoallelic and multihit TP53 alterations is therefore critical for prognosis and treatment planning. The current diagnostic criteria by International Consensus Classification (ICC) classify cases with 10-19% blasts with any TP53 mutation with VAF >10% as high-risk myeloid neoplasms, regardless of whether they meet classic AML or MDS thresholds. Recent updates by both the World Health Organization (WHO, 5^th^ edition) and ICC emphasize the poor prognosis of biallelic/multihit TP53 mutations in MDS/AML. Such cases are characterized by one or more of the following: two or more TP53 mutations each with a variant allele frequency (VAF) above 10%, VAF ≥ 50%, loss of the wild-type TP53 allele (e.g., 17p deletion or monosomy 17), or the presence of a complex karyotype (5).

Moreover, higher VAF levels have been correlated with poorer survival outcomes. A multicenter study involving 359 patients with complex karyotype MDS found that those with TP53 mutations and VAF > 40% had a median overall survival of only 0.6 years, significantly lower than those with VAF <40% (1.1 years, p=0.004). Even patients with VAF <40% had worse outcomes than TP53 wild-type patients (1.1 vs 1.5 years, p= 0.001) (8, 58). These findings highlight the prognostic impact of VAF and support its integration into future diagnostic and treatment algorithms.

Given the strong association between TP53 mutations, genomic instability, and disease evolution, clonal hematopoiesis provides important insight into early leukemogenic events in TP53-mutated MDS and AML. Clonal hematopoiesis is age-associated expansion of hematopoietic cells driven by somatic mutations. Despite small size of the clones, these mutations lead to increased fitness of mutated clones, posing an increased risk of leukemic transformation affecting 0.5-1% carriers/year, particularly TP53 and U2AF1 (59). There is convincing evidence that TP53 mutations in individuals with clonal hematopoiesis of indeterminate potential (CHIP) are associated with increased risk of hematological cancers (60–63). TP53 mutation is also seen more frequently in CHIP patients who have received prior radiotherapy or cytotoxic chemotherapy (64). Gene mutations like DNMT3A, TET2, ASXL1, SRSF2, CBL and SF3B1 with TP53 mutation are associated with higher risk of leukemia (15, 39, 65). A recent study showed TP53, NPM1 and SRSF2 as dominant mutations preceding the AML whereas DNMT3A and TET2 mutations were stable over time (61). Therefore, understanding the correlation between clonal hematopoiesis and mechanisms of progression into leukemia remains crucial to design therapies targeting TP53 mutation.

### Standard chemotherapy in TP53-Mutated MDS/AML

For younger and fit patients with AML, standard induction regimens traditionally include cytarabine in combination with an anthracycline, such as the “7+3” regimen or the FLAG-Ida protocol (fludarabine, cytarabine, idarubicin, and G-CSF) (66). However, the patients harboring TP53 mutations consistently demonstrate poor response to standard therapies, including intensive chemotherapy and allogenic hematopoietic stem cell transplantation (allo-HSCT). Outcomes are particularly unfavorable in cases with biallelic TP53 mutations and complex or monosomal karyotypes (CK/MK) (67, 68). Multivariant analysis showed that TP53 mutations are independently associated with inferior overall outcomes (OS), reduced disease-free survival (DFS), and lower response rates (RR), irrespective of underlying cytogenetic abnormalities (69). Relapse rates following induction may reach up to 20-30% (53, 70), with a median OS in the range of 4–9 months, underscoring the limited efficacy of conventional cytotoxic regimens in this high-risk population (71–74). **Table 1** details outcomes of TP53-mutated AML and MDS in several clinical trials. **Table 1** following standard chemotherapy.

**Table 1:** Key clinical trials for TP53-mutated AML and MDS.

NCT#	Year	Phase	Patients	Interventions	N (TP53m)	No. of mono- vs bi-allelic mutation	Responses (TP53m vs WT)	Median OS, mon (TP53m vs WT)
NCT01687400 (75)	2016	II	MDS or AML	Decitabine	116 (21)	20;1	ORR: 100% vs. 41%	12.7 vs. 15.4
NCT02397720 (76)	2019	II	AML	Azacitidine + Nivolumab	70 (16)	NR	ORR: 19% vs. 33%	5.98 vs. 6.60
NCT03404193 (77)	2020	II	AML	Decitabine + Venetoclax	168 (31)	30;1	CR/CRi: 69% vs. 81%	6.9 vs. 18.1
NCT02152956 (78)	2021	I/II	AML	Flotetuzumab	88 (1)	NR	ORR: 30.0 vs NR	4.0 vs. 11.2
NCT03588078 (79)	2021	II	MDS or AML	Eprenetapopt + Azacitidine	52 (52)	31;21	ORR 52%, CR 37%	12.1 (MDS); 10.4 (AML)
NCT03072043 (80)	2021	II	MDS or AML	Eprenetapopt (APR-246) + Azacitidine	55 (55)	6;49	ORR: 71.0% vs. NR	10.8 vs. NR
ISRCTN78449203 (81)	2023	III	MDS or AML	CPX-351 vs FLAG-Ida	189 (81)	NR	NR	7.0 vs. 28.0
NCT03248479 (82)	2023	Ib	MDS	Magrolimab+ Azacitidine	95 (25)	NR	ORR: 68.0% vs. 78.7%	16.3 vs. not reached
NCT04214860 (83)	2023	I	AML	eprenetapopt + venetoclax + azacitidine	49 (49)	9;40	ORR: 64% vs. NR	NR
NCT03113643 (84)	2024	Ib/II	AML	Tagraxofusp + Azacitidine + Venetoclax	26 (13)	4;9	ORR: 54% vs. 85%	9.5 vs. not reached
NCT03946670 (85)	2024	II	MDS	Sabatolimab + HMA vs Placebo + HMA	127 (47)	NR	ORR 68% (Sabatolimab + HMA) vs. 61% (Placebo + HMA)	19.0 (Sabatolimab + HMA) vs. 18.0 (Placebo + HMA)
NCT03214562 (86)	2025	II	AML	FLAG-Ida + Ven	138 (6)	5;1	ORR 97% vs. 100% (ND); 52% vs. 79% (R/R)	13 vs. not reached (ND); 9 vs. not reached (R/R)
NCT03588078 (87)	2025	II	MDS or AML	Eprenetapopt (APR-246) + Azacitidine	100(100)	12:88	ORR 69%; CR 41%	11.8

*CPX-351, Liposomal encapsulated daunorubicin and cytarabine; 7+3, 7 days of cytarabine + 3 days of daunorubicin; FLAG-Ida, Fludarabine, cytarabine, idarubicin, and G-CSF; WT, wild-type; TP53m, TP53-mutated; DOR, duration of response; HMA, decitabine or azacitidine; ND, newly diagnosed; R/R, relapsed or refractory.

#### CPX-351 in TP53-mutated AML

CPX-351 is a liposomal formulation of danorubicin and cytarabine approved for the treatment of therapy-related AML and AML with myelodysplasia-related changes, clinical entities in which TP53 mutations are commonly enriched. Although CPX-351 has demonstrated improved outcomes in secondary AML compared with conventional 7+3, its benefit in TP53-mutated disease appears limited (81). Patients with TP53-mutated AML treated with CPX-351 consistently show lower remission rates, early relapse, and short overall survival, particularly in the presence of multihit TP53 alterations and complex karyotypes (6, 81). Overall, outcomes in this subgroup appear comparable to those achieved with other intensive cytotoxic regimens and remain substantially inferior to those observed in TP53-wild type AML, indicating that CPX-351 does not overcome TP53-associated chemoresistance (28).

#### Hypomethylating agents

Hypomethylating agents azacitidine and decitabine are the standard first-line treatments for elderly AML patients and those with high-risk MDS (HR-MDS). Azacitidine is a cytidine analog that after incorporation into DNA acts as a noncompetitive inhibitor of DNA methyltransferase 1 (DNMT1) (88). In the AZA-001 phase III trial, azacitidine was compared with conventional care regimens (best supportive care, low-dose cytarabine, or intensive chemotherapy) in patients with higher-risk myelodysplastic syndromes. Azacitidine significantly prolonged overall survival (median 24.5 vs 15.0 months; HR 0.58), delayed progression to acute myeloid leukemia and was associated with higher rates of hematologic response compared with conventional care (89). In the VIALE-A trial, which compared azacitidine plus venetoclax with azacitidine alone in previously untreated AML patients ineligible for intensive chemotherapy, azacitidine monotherapy was associated with median overall survival of 9.6 months and a composite complete remission (CRc) rate of 28.3% in the overall study population. However, outcomes in the TP53-mutated subgroup were particularly poor, with no observed remissions (CRc 0%) and a median overall survival of approximately 5–6 months, highlighting limited clinical benefit of single agent azacitidine in this high-risk molecular subset (90).

Decitabine, a deoxycytidine analog, functions by causing widespread hypomethylation, restoring expression of silenced tumor suppressors, and induction of DNA damage. Decitabine is currently FDA-approved for the treatment of MDS. However, it has not received FDA approval for AML, as a large randomized international phase III trial comparing decitabine with supportive care and low-dose cytarabine in elderly AML patients demonstrated improved complete remission rates but no statistically significant benefit in overall survival (91). In contrast, decitabine is approved by the European Medicines Agency and is recommended by the NCCN guidelines. Despite the lack of FDA approval, it continues to be used off-label for AML in the United States (92). The clinical trial with 10-day decitabine reported a 100% response rate (21/21) among TP53 mutated cases treated with 10-day decitabine regimen compared to 41% (32/78) in TP53 wild-type patients (p < 0.001) (75). Median overall survival was 12.7 months in patients with TP53 mutations and 15.4 months in those with wild-type TP53 (p = 0.79) (75). Importantly, these findings were derived from a non-randomized, single-institution study and have not been consistently reproduced in subsequent trial. For example, in a single institution phase II trial comparing 5-day and 10-day decitabine (DAC) regimens in TP53-mutated AML, response rates (29% vs 47%, p = 0.40) and overall survival (5.5 vs 4.9 months; p = 0.55) were not significantly different between regimens (93). A separate phase 3 trial demonstrated superior complete remission (CR) rates with decitabine (17.8%) compared to standard care (7.8%) in older AML patients with poor/intermediate-risk cytogenetics (p = 0.001). Median overall survival also favored decitabine (7.7 months vs. 5.0 months), although the difference did not reach statistical significance (91). Notably, reduction in TP53 variant allele frequency (VAF <5%) during HMA treatment has been associated with improved survival and is increasingly used as a prognostic biomarker, particularly in patients bridged to allo-HSCT (58, 94, 95). Although baseline VAF does not consistently predict response, VAF ≥ 40% has been linked to inferior outcomes (median OS 4.1 vs 7.7 months with HMA therapy) (94, 95). While a 10-day monthly decitabine regimen (DEC10) has produced encouraging blast clearance but mutation reduction responses are not durable, necessitating consolidating strategies such as transplantation (75).

#### Venetoclax

Venetoclax, a BCL-2 inhibitor, promotes apoptosis by targeting anti-apoptotic proteins in leukemic blasts (96, 97). It was initially promising in the TP53-mutated AML, as BCL-2 dependence was thought to be independent of p53 function (98, 99). Venetoclax in combination with low intensity therapies (HMA or low dose cytarabine) gained regulatory approval for older or unfit patients with AML in 2022 (90). Subsequent studies showed limited benefit of venetoclax in TP53-mutated patients, especially those with multi-hit TP53 alterations. In a prospective phase trial evaluation 10-day decitabine plus venetoclax in elderly AML, TP53-mutated patients had a median OS of only 5.3 months, significantly shorter than TP53 wild-type patients (19.4 months; HR 4.67, p<0.00001), with lower MRD clearance and higher early mortality (100). A large retrospective analysis of 238 patients with newly diagnosed TP53 mutation AML receiving venetoclax-based therapy found no significant difference in OS (6.6 vs 5.7 months, p= 0.4) or relapse free-survival (4.7 vs 3.5 month; p=0.43) when compared to non-venetoclax-based regimens (101).

Venetoclax has been studied in combination with intensive chemotherapy regimens such as FLAG-Ida and 7+3. In a phase 1b trial, 34 patients with newly diagnosed AML received standard 7+3 induction (daunorubicin plus cytarabine) alongside venetoclax administered for escalating durations (8, 11, or 14 days) (102). The combination was well tolerated in fit adults up to 75 years of age, with no induction mortality, and achieved high efficacy (composite complete remission in 85.3% of patients and MRD negativity in 86%). Responses were consistent across most ELN 2022 risk groups, with the notable exception of the TP53-mutated subgroup (5 of 34 patients), where remission durability and survival outcomes remained poor (102).

Similarly, a recent study of intensive chemotherapy plus venetoclax (NCT03214562), 138 AML patients (77 newly diagnosed, 61 relapsed/refractory) receiving FLAG-IDA (Fludarabine, cytarabine, granulocyte colony-stimulating factor (G-CSF), and idarubicin) plus venetoclax achieved a 97% overall response rate, with 95% of patients attaining a composite complete remission and 90% clearing measurable residual disease by flow cytometry (86). Two-thirds remained alive and event-free at three years. Importantly, the study reported six newly diagnosed patients harboring TP53 mutations (two with VAFs < 5%; one bi-allelic with VAFs 20% and 22%; and three with VAFs 17%, 34% and 54%), all achieved an MRD-negative CRc, but remissions were short with median duration of response 8 months. Five of six subsequently relapsed and died, with median overall survival of 13 months (86). These results highlight that even the most active regimens may offer only transient benefit in multi-hit TP53-mutated AML, underscoring the urgent need for novel strategies in this high-risk subgroup.

#### Allogeneic hematopoietic stem cell transplant (allo-HSCT)

Allogeneic hematopoietic stem cell transplant (allo-HSCT) remains the only potentially curative option for patients with TP53-mutated AML and MDS. However, outcomes are poor, with relapse and mortality rates significantly higher, up to 80-90% compared to TP53 wild-type patients (103–105). In a recent retrospective analysis of 240 patients with TP53-mutated AML or MDS undergoing allogeneic HSCT from matched related, matched unrelated, or haploidentical donors, TP53 variant allele frequency (VAF) with cytogenetic features stratified post-transplant outcomes (106). TP53 VAF and cytogenetics were the strongest predictors of outcome, defining three prognostic groups: patients with TP53 VAF ≥50% had a 2-year PFS of 3%; those with TP53 VAF <50% and complex cytogenetics or del(5q)/del(7q) had a 2-year PFS of 22%; and patients with TP53 VAF <50% without these abnormalities achieved a favorable 2-year PFS of 60% (106). Such data suggests that TP53-mutated AML/MDS should not be treated as a single high-risk entity.

Several barriers limit transplant success in this population (1): Older age and comorbidities often necessitate reduced-intensity conditioning compromising MRD clearance (107) (2); Outcomes are influenced more by baseline TP53 status than by mutational burden at remission (108) (3); Rapid disease progression in TP53-mutated AML often negates the graft-versus leukemia (GVL) effect, diminishing transplant efficacy (109). Importantly, data suggest that patients transplanted in CR may derive significant benefit, with reduced relapse and mortality risk (110). In a prospective study comparing allogeneic SCT with donor versus no-donor control group in patients with high-risk MDS (111), overall survival was significantly higher at four years in patients with a donor compared to those without (37% vs 15%), and disease-related mortality was lower (37% vs 73%). Notably, the survival advantage became evident only after the second year, reflecting early transplant-related mortality (111). Therefore, early identification of the transplant-eligible patients achieving deep remissions remain critical goals.

#### Newer therapies (future perspective)

**Figure 2:**
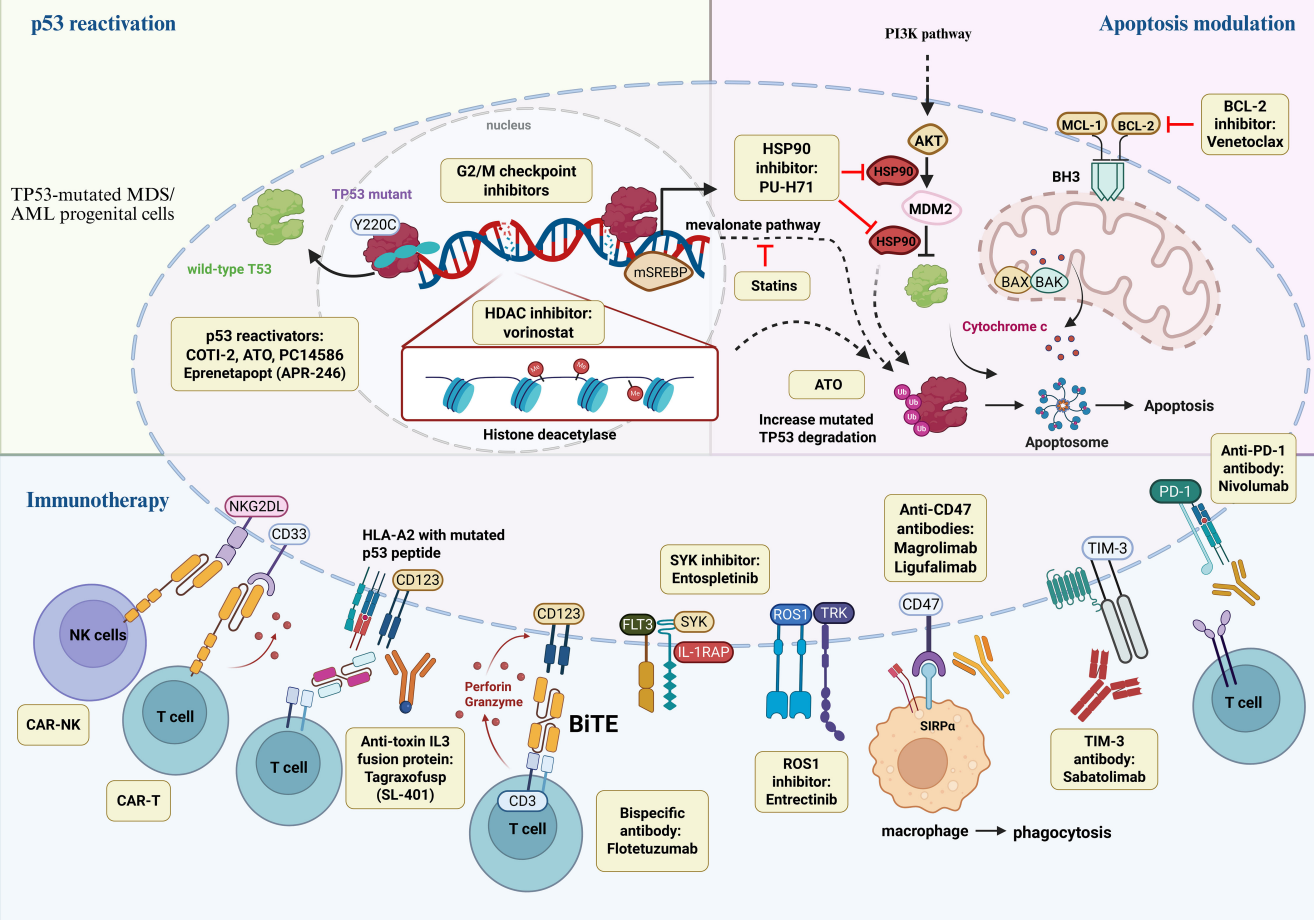
Therapeutic landscape in TP53-mutated AML. This schematic summarizes the rapidly evolving therapeutic landscape in TP53-mutated AML, organized into three mechanistic categories. (1) p53 reactivation aims to restore or modulate aberrant p53 signaling via direct p53 reactivators (e.g., eprenetapopt, COTI-2); (2) Apoptosis modulation through intrinsic mitochondrial pathways, including BCL-2 family inhibition (e.g., venetoclax), promotion of mutant TP53 degradation (e.g., statins) and modulation of complementary survival signals (e.g. SYK); (3) Immunotherapeutic strategies engage macrophage-mediated phagocytosis (anti-CD47 antibodies such as magrolimab), T-cell–based therapies (BiTE and anti-PD-1 and anti-TIM-3 antibodies), antibody–drug or toxin conjugates (e.g., tagraxofusp targeting CD123), and cellular therapies (e.g. CAR-T). ATO, arsenic trioxide; BCL-2, B-cell lymphoma 2; BAX, BCL-2–associated X protein; BAK, BCL-2 antagonist/killer; BiTE, bispecific T-cell engager; CAR, chimeric antigen receptor; CD, cluster of differentiation; HDAC, histone deacetylase; HSP90, heat shock protein 90; IL-1RAP, interleukin-1 receptor accessory protein; MCL-1, myeloid cell leukemia 1; MDM2, mouse double minute 2; NK, natural killer; PD-1, programmed cell death protein 1; PI3K, phosphoinositide 3-kinase; SYK, spleen tyrosine kinase; TIM-3, T-cell immunoglobulin and mucin-domain containing-3; TCR, T-cell receptor; TRK: tropomyosin receptor kinase; mSREBP: mature sterol regulatory element-binding proteins.

Given the limited efficacy of current treatments, novel therapies targeting TP53-mutated AML/MDS are under active investigation and may serve as effective bridging or maintenance strategies after transplant. A schematic overview of emerging therapeutic strategies in TP53-mutated AML/MDS is shown in **Figure 2**.

Targeted therapies.

Therapeutic approaches in TP53-mutated AML/MDS include:

- Reactivation of mutant p53: Agents such as Eprenetapopt (APR-246) and COTI-2 restore wild-type conformation and function through covalent binding or refolding mechanisms (112–115)
- Synthetic lethality: G_2_/M checkpoint inhibitors (e.g., CHK1/CHk2, WEE1, PLK1 inhibitors) selectively kill TP53-deficient cells by overriding DNA-damage arrest (116–118)
- Mutant p53 degradation: Statins, HSP90 inhibitors (e.g., PU-H71), and HDAC inhibitors (e.g., vorinostat) induce p53 degradation via proteasome or chaperone disruption (119–124)
- Kinase pathway blockade: Syk inhibitors (entospletinib) and ROS1 inhibitors (entrectinib) aim to inhibit leukemogenic survival signaling (125–127)
- Arsenic trioxide (TO): Rescues wild-type conformation in specific TP53 mutants via cysteine targeting, and may induce ferroptosis (128)

**Table 2** below highlights selected targeted therapies in TP53-mutated AML and MDS.

**Table 2:** Potential targets and therapeutic agents in TP53-mutated AML and MDS.

Class	Examples	Mechanism of action	Ref.
HSP-90 inhibitors	PU-H71 (zelavespib)	Disrupts epichaperome complexes	(129)
Statins	Atorvastatin	Promotes proteasomal degradation of misfolded mutant p53 proteins	(121–123)
HDAC inhibitors	Vorinostat	Enhances histone acetylation, modulating transcriptional activity to induce apoptosis in malignant cells	(124)
Syk inhibitors	Entospletinib	Suppresses aberrant SYK tyrosine kinase signaling, which contributes to leukemogenesis	(125)
ROS1 inhibitor	Entrectinib	Inhibits ROS1-driven receptor tyrosine kinase signaling involved in survival and proliferation of leukemic cells	(126, 127)
Mutant TP53 refolding	Arsenic trioxide	Restores wild-type p53 conformation and function by stabilizing the DNA-binding domain of mutant p53	(128)

## Eprenetapopt (APR-246)

Eprenetapopt (APR-246) is a small molecule that targets mutant p53 by converting mutant p53 back to its wild-type conformation and function. Chemically, it is a methylated analogue of PRIMA-1, which is spontaneously converted into the active metabolite methylene quinuclidinone (MQ). MQ targets the thiol groups of cysteines in mutant p53, creating covalent links that refold the protein and reactivate its tumor‐suppressor role (113, 114). Preclinical data suggest additional mechanism including oxidative stress induction, ferroptosis, and dNTP depletion (130–133). Combination with azacitidine demonstrated synergy *in vitro* (134).

In a phase Ib/II trial, eprenetapopt + azacitidine produced CR rates of 50% in TP53-mutant MDS and 36% in AML, with molecular remissions (TP53 VAF <5%) in 38% of responders. Bridging to allo-HSCT in molecular responders lead to improved survival (OS not reached vs 9.1 months) (80). Despite promising early-phase data, subsequent trials yielded more modest results. A triplet regimen (eprenetapopt + azacitidine + venetoclax) showed a CR/CRi of 53% and a median OS of 8 months, similar to HMA-VEN alone in newly diagnosed AML patients. A phase II post-transplant maintenance study reported a median relapse-free survival (RFS) of 12.5 months and a median OS of 20.6 months, although these data were derived from small, non-randomized cohorts (135). Recently, the long-term follow-up of the aforementioned phase 2 trials (80, 132) was published (87). By intention-to-treat, the overall response rate was 69%, including 41% CR, with TP53 clearance (VAF <5%) achieved in 40%. Median CR duration was 10.6 months and median OS 11.8 months. While outcomes were markedly better in patients achieving CR or TP53 clearance before allogeneic transplantation (87), eprenetapopt-based therapy has not consistently resulted in durable remission or long-term survival benefit in TP53-mutated AML/MDS.

COTI-2, a third-generation thiosemicarbazone, also restores p53 function by binding to mutant p53 and reactivating its tumor-suppressive transcriptional activity. In addition to p53 reactivation, it exhibits p53-independent effects, including DNA damage induction, AMPK activation, and mTOR pathway inhibition (114, 115, 136). Preclinical studies have shown activity in TP53-mutant solid tumors, and early-phase clinical trials in gynecologic and head and neck cancers have reported acceptable safety profiles (136, 137). Other agents and small molecules that show promises include: CP-31398: a p53 refolding molecule (138); RITA: disrupts the p53-MDM2 interaction to restore p53 activity (139); PK110007 (140); HO-3867, a STAT 3 inhibitor (141); and PK7088 (142).

### CD47 targeting agents: magrolimab (Hu5F9-G4)

CD47 is a transmembrane “don’t eat me” signal overexpressed on both healthy and malignant hematopoietic cells, including leukemic stem cells (LSCs), where it binds signal regulatory protein α (SIRPα) on macrophages to inhibit phagocytosis (143, 144). Magrolimab, a first in class humanized anti-CD47 IgG4 monoclonal antibody, showed initial promising results in combination with azacitidine in TP53-mutated AML and MDS, with early-phase studies reporting response rates of 40-71% and median overall survival of up to 16.3 months (144, 145). However, results from three randomized phase III trials- ENHANCE (MDS), ENHANCE-2 (TP53-mutant AML), ENHANCE-3 (unfit AML) - failed to show survival benefit. In ENHANCE, the magrolimumab arm has 139 deaths versus 124 in the control group, with a hazard ratio (HR) for OS of 1.254 (95% CI, 0.980-1.605), leading to discontinuation (146, 147). In ENHANCE-2, the median OS with magrolimumab plus azacitidine was 4.4 months versus 6.6 months with azacitidine alone (HR 1.13; 95% CI, 0.78-1.64) (146–148). Similarly, in ENHANCE-3, patients with previously untreated AML who were ineligible for intensive chemotherapy received either magrolimab or placebo, each in combination with venetoclax and azacitidine. It failed to improve OS (10.7 vs 14.1 months, HR 1.18; 95% CI, 0.85-1.64) or remission rates compared to placebo (149). As of 2024, development of magrolimab in hematologic malignancies has been discontinued, and the FDA has placed a full clinical hold on all related trials.

### Ligufalimab (AK117)

Ligufalimab (AK117) is a second-generation humanized IgG4 anti-CD47 antibody. Unlike magrolimab, ligufalimab does not cause red blood cell agglutination, even at high concentrations. It has been shown to induce only mild, transient anemia in non-human primates (150). Preclinically, it showed strong phagocytic activity and tumor inhibition in xenograft models. Notably, AK117 eliminates the need for a priming dose, increased hemoglobin thresholds, or additional monitoring (150). In a phase 1b trial in newly diagnosed higher-risk MDS (n=72; median age 66), ligufalimab plus azacitidine achieved a 48.1% CR rate with manageable safety profile (151). Anemia occurred in 29.1% of patients, grade ≥3 in 24.4%, with 61.5% of transfusion-dependent patients achieved independence (151). Given such promising results, a phase 2 study (NCT06196203) is currently evaluating ligufalimab with azacitidine in 90 higher-risk MDS patients in the U.S. and China, randomized 1:1:1 to ligufalimab 30 mg/kg every two weeks, ligufalimab 20 mg/kg every two weeks, or placebo (152). The primary endpoint is complete remission rate (CRR) per International Working Group (IWG) 2023, with secondary endpoints including overall response rate (ORR), duration of response (DoR), event-free survival (EFS), overall survival (OS) and others (152).

### Sabatolimab

T-cell immunoglobulin and mucin domain 3 (TIM-3) is an inhibitory checkpoint that is expressed on leukemic stem cells, blasts and IFN-γ producing T lymphocytes, particularly in TP53-mutant disease. MDS/AML cells overexpress TIM-3 and its ligand galatectin-9 which forms an autocrine signaling loop to enhance leukemic stem cells (LSCs) maintenance (153–155). Sabatolimab (MBG453) is a humanized, IgG4 antibody targeting TIM-3 on myeloid cells which eliminates AML LSCs. In a phase Ib trial evaluated sabatolimab and azacitidine in unfit AML or HR-MDS, ORR was 71.4% in MDS and CR/CRi rate of 40% in newly diagnosed mutated AML (156). However, a subsequent randomized phase II STIMULUS-MDS1 trial did not meet its primary endpoints, with no significant improvement in complete remission rate or progression-free survival compared to hypomethylating agents alone (CR 22% vs. 18%, p=0.77; PFS 11.1 vs. 8.5 months, p=0.1022) compared to placebo (85). Taken together, these findings indicate that despite a strong biological rationale and early-phase activity, sabatolimab has not demonstrated clear clinical benefit in randomized studies.

### Immune checkpoint inhibitors-based regimens

Binding of the programmed death-1 (PD-1) receptor to its ligand PD-L1 produces immunosuppressive microenvironment which is hijacked by the leukemic blasts for unrestricted proliferation (157). It was reported that AML blasts with TP53 mutations had augmented upregulation of PD-1 which was also seen in patients treated with HMAs (158–161). This led to an interest in combination therapies to overcome the resistance. However, the clinical outcomes have been variable and overall limited.

A phase 2 combination study of nivolumab and 5-azacitidine reported a 33% ORR overall, and 52% among patients who had not previously received HMAs. Additionally, factors like presence of CD3+ infiltrate, ASXL1 mutation, lower burden (<20% BM blasts) were associated with improved overall response rates (76). However, a recent randomized phase 2 trial for HR-MDS and older patients with AML of azacitidine with or without durvalumab as first line therapy showed similar overall response rates (162). Similarly, combination of anti-cytotoxic T-cells CTLA-4 antibody (ipilimumab) to 5-AZA and nivolumab did not show significant difference when compared to venetoclax + 5-AZA or 5-AZA + nivolumab (163). Overall, immune checkpoint inhibitor-based strategies have not demonstrated a consistent improvement in overall survival in TP53-mutated AML or MDS.

### CAR-T

Chimeric antigen receptor T cell therapy (CAR-T) emerged as a novel therapy with dramatic response rate in relapsed B-cell malignancies and multiple myeloma (164–171). This led to studies to determine the efficacy of CAR-T in patients with AML/MDS, especially TP53 mutated patients. The key element would be to direct the CAR-T cells against specific myeloid antigen like CD33, CD38, CD70, CD117, CD123, CD371, CLL1, FLT3, and others. Unfortunately, functional TP53 loss in AML confers resistance to CAR T-cell therapy via longer interaction period between CAR T-cells with TP53-deficient AML cells, which causes T-cell exhaustion and reduced CAR-T proliferation ultimately leading to leukemic cell expansion (164). It is known that CTLs and natural killer (NK)-cells apoptosis is p53-mediated on the target cells (172–175). Interestingly, the mevalonate pathway was upregulated through transcriptional profiling of the TP53-mutated AML cells under the CAR-T cell therapy. Furthermore, the effect of simvastatin, an HMG-CoA reductase inhibitor that blocks the rate-limiting step in cholesterol synthesis, was observed as a rescue mechanism for CAR-T cell therapy (164). Additionally, ineffective activation of Wnt pathway, which is essential for development, differentiation and survival of mature T lymphocytes may contribute to the CAR-T cell resistance in the TP53 deficient AML cells (164). These findings offer potential pathways to overcome the resistance of the TP53 mutated AML cells to CAR-T cell therapy.

## Other novel approaches

### TP53-Y220C PC14586

Y220C is a common hotspot mutation, seen in approximately 1% of all solid cancers and constitutes <5% of all mutations in TP53m-AML (15). This mutation creates a surface crevice that leads to an unstable p53 structure and drives carcinogenesis (176–178). PC14586 (PMV Pharmaceuticals) was designed as the first orally bioavailable, reactivator of p53 Y220C mutant. It binds to Y220C p53 which results in stabilization of the molecule, reactivating the transcription mediated cell-cycle arrest and apoptosis. The preliminary efficacy was seen in patients with solid cancers who achieve partial responses and stable disease in an ongoing phase I trial (179). It was also reported that combination therapies with XPO-1, MDM2 or Bcl-2 inhibitors leads to upregulation of PC14586-induced p53 target proteins causing massive apoptosis (129). Further trials are needed to determine the efficacy and durability of this novel therapy.

### Arsenic trioxide (ATO)

ATO is a drug used for the treatment of acute promyelocytic leukemia (180). Structural studies have revealed that ATO binds a cryptic allosteric site composed of three cysteine residues within the p53 DNA-binding domain and helps stabilize certain misfolded TP53 mutants and partially restores function (181). Further mechanistic studies using AML cell-line models demonstrated that ATO also induces ferroptotic cell death in TP53-mutant cells by depleting glutathione peroxidase 4 (GPX4) and the cystine transporter SLC7A11 (182). An ongoing Phase I trial in China (NCT03855371) is evaluating the safety and efficacy of decitabine in combination with intravenous ATO in patients with high-risk MDS harboring TP53 mutations. The study prioritizes selection of high-risk TP53-mutant MDS patients with predicted sensitivity to the combination, informed by prior mechanistic studies. A separate ongoing Phase II study (NCT06778187) is investigating oral arsenic trioxide combined with ascorbic acid and investigator-selected low-intensity therapy in previously untreated or relapsed/refractory TP53-mutated AML, MDS, or CMML. The backbone therapy includes a hypomethylating agent e.g. azacitidine, decitabine, or decitabine-cedazuridine with or without venetoclax. The results of tolerability and response data are yet to be reported.

### Bispecific antibodies

Flotetuzumab is a CD123×CD3 bispecific DART (dual-affinity retargeting) antibody that facilitates T-cell activity against CD123-expressing AML blasts. This facilitates T-cell redirection and activation, leading to targeted lysis of AML cells. CD123 subunit is highly expressed in over 90% of AML blasts and leukemic stem cells particularly in adverse-risk subgroups. Upon binding, Flotetuzumab triggers dose-dependent T-cell-mediated cytotoxicity and cytokine production (183).

In a phase I/II study (CP-MGD006-01), Flotetuzumab was evaluated in relapsed/refractory AML. Among 22 patients, 15 had TP53 mutations and/or 17p deletions. In this subgroup, a CR/CRi rate of 47% was observed, with a median OS of 10.3 months (183). Notably, data showed that flotezumab upregulated the major histocompatibility class II (MHC-II) in AML via JAK-STAT signaling, particularly mediated by the local production of interferon-γ. These findings suggest that T-cell immunotherapy can target relapsed AML, particularly in patients with immune-infiltrated disease (184, 185).

Another agent tagraxofusp (SL-401), anti-toxin IL3 fusion protein against CD-123 which was approved for blastic plasmacytoid dendritic cell neoplasm, was tested in clinical trials for relapsed/refractory CD123-positive AML (NCT04342962). Preliminary findings indicate that tagraxofusp produced an overall response rate of 34.8% in relapsed/refractory CD123-positive AML, with capillary leak syndrome occurring in 21.7% of patients (186). A recent phase 1b trial evaluated tagraxofusp in combination with azacytidine with or without venetoclax in AML (84). Among 26 patients with adverse-risk AML, 50% (13/26) carried TP53 mutations, most of which were multi-hit (9/13). In this subgroup, the overall response rate was 54% (7/13), and 57% (4/7) of responders achieved MRD negativity by flow cytometry. Median overall survival was 9.5 months (95% CI, 1.8–NA), notably longer than the ~5 months typically reported in poor-risk TP53-mutated AML treated with AZA-VEN alone. Median progression-free survival was 5.1 months (95% CI, 1.8–NA) (84).

Several ongoing clinical trials are further investigating tagraxofusp-based regimens, including its combination with low-intensity chemotherapy in relapsed/refractory AML (NCT06561152), with or without VEN in newly diagnosed secondary AML (NCT05442216) and with VEN + AZA in untreated AML ineligible for intensive therapy (NCT06456463).

Another innovative strategy targets the common TP53 hotspot mutation R175H as a therapeutic neoantigen (187). A CD3 bispecific antibody was engineered to recognize the mutant p53R175H peptide presented by HLA-A*02:01, redirecting T-cell activity against tumor cells. Despite the very low density of peptide–HLA complexes on the cell surface, this bispecific antibody triggered robust cytokine release and selective lysis of R175H-mutant cancer cells *in vitro*, without off-target activity, and suppressed or regressed tumors in NSG mice engrafted with human T cells (187). These findings provide the first proof-of-concept that TP53 neoantigens can be effectively targeted with antibody-based immunotherapy.

## Conclusion

In summary, TP53-mutated MDS and AML represent a clinically distinct, high-risk subgroup marked by complex karyotypes, chemoresistance, and dismal outcomes. Somatic TP53 alterations disrupt the p53 tumor-suppressor network, abrogating its capacity to enforce cell-cycle arrest, DNA repair, and apoptosis under stress. As a result, standard cytotoxic regimens (7+3, FLAG-Ida), hypomethylating agents ± venetoclax, and even allogeneic stem-cell transplantation yield suboptimal response rates and short median survival (6–12 months). Although emerging therapeutic strategies aimed at restoring p53 function, modulating epigenetic programs, or engaging immune-based mechanisms have generated considerable interest, their clinical benefit remains investigational without a clear, reproducible survival advantage. This underscores the continued unmet need for novel therapeutic approaches, innovative trial designs and analysis of long-term outcome data.
