# Correction: TP53-mutated myelodysplastic syndromes and acute myeloid leukemia: a comprehensive overview of targeted approaches

**DOI:** 10.3389/fonc.2026.1823072

**Published:** 2026-03-13

**Authors:** Shweta Deshpande, Wing Fai Li, Junmin Song, Mark Forsberg, Claudio Cerchione, Giovanni Martinelli, Marina Konopleva

**Affiliations:** 1 Department of Oncology, Montefiore Einstein Comprehensive Cancer Center, Bronx, NY, United States; 2 Department of Medicine, Jacobi Medical Center, Albert Einstein College of Medicine, Bronx, NY, United States; 3 Department of Hematology and Oncology, IRST IRCCS, Meldola, Italy; 4 Department of Medical and Surgical Sciences, Università di Bologna, Bologna, Italy; 5 Department of Cell Biology, Albert Einstein College of Medicine, Bronx, NY, United States

**Keywords:** acute myeloid leukemia, myelodysplastic syndromes, TP53 mutation, p53, hypomethylating agents, venetoclax, allogeneic stem cell transplantation

The title of this article was erroneously given as: “TP53-mutated myelodysplastic syndromes and acute myeloid leukemia: a 3 comprehensive overview of targeted approaches.” The correct title of the article is “TP53-mutated myelodysplastic syndromes and acute myeloid leukemia: a comprehensive overview of targeted approaches.”

The original version of this article has been updated.

